# Bigger Helpers in the Ant *Cataglyphis bombycina*: Increased Worker Polymorphism or Novel Soldier Caste?

**DOI:** 10.1371/journal.pone.0084929

**Published:** 2014-01-03

**Authors:** Mathieu Molet, Vincent Maicher, Christian Peeters

**Affiliations:** 1 Laboratoire Ecologie & Evolution – Unité Mixte de Recherche 7625, Université Pierre et Marie Curie, Paris, France; 2 Laboratoire Ecologie & Evolution – Unité Mixte de Recherche 7625, Centre National de la Recherche Scientifique, Paris, France; Smithsonian Conservation Biology Institute, United States of America

## Abstract

**Introduction:**

The mechanisms by which development favors or constrains the evolution of new phenotypes are incompletely understood**.** Polyphenic species may benefit from developmental plasticity not only regarding ecological advantages, but also potential for evolutionary diversification. For instance, the repeated evolution of novel castes in ants may have been facilitated by the existence of alternative queen and worker castes and their respective developmental programs.

**Material and Methods:**

*Cataglyphis bombycina* is exceptional in its genus because winged queens and size-polymorphic workers occur together with bigger individuals having saber-shaped mandibles. We measured seven body parts in more than 150 individuals to perform a morphometric analysis and assess the developmental origin of this novel phenotype.

**Results:**

Adults with saber-shaped mandibles differ from both workers and queens regarding the size of most body parts. Their relative growth rates are identical to workers for some pairs of body parts, and identical to queens for other pairs of body parts; critical sizes differ in all cases.

**Conclusions:**

Big individuals are a third caste, i.e. soldiers, not major workers. Novel traits such as elongated mandibles are combined with a mix of queen and worker growth rates**.** We also reveal the existence of a dimorphism in the queen caste (microgynes and macrogynes). We discuss how novel phenotypes can evolve more readily in the context of an existing polyphenism. Both morphological traits and growth rules from existing queen and worker castes can be recombined, hence mosaic phenotypes are more likely to be viable. In *C. bombycina*, such a mosaic phenotype appears to function both for defense (saber-shaped mandibles) and fat storage (big abdomen). Recycling of developmental programs may have contributed to the morphological diversity and ecological success of ants.

## Introduction

During animal ontogeny, organs follow distinct growth rules that produce an integrated phenotype. Two parameters are crucial: growth rate defines the speed at which each organ grows, and critical size specifies the overall size of the individual at which growth stops and development is complete [1]. By modifying growth rules (growth rate and critical size), new phenotypes can be produced. Phenotypic plasticity relies on such modifications to generate variation. Changing critical size without altering growth rates results in new phenotypes that are in the continuity of existing ones. Potential changes are thus limited. In contrast, modifying growth rates can lead to dramatically different phenotypes. Here we explore how growth rules are modified to generate new phenotypes by using one of the most polyphenic animal taxon as a model: ants.

**Figure 1:**
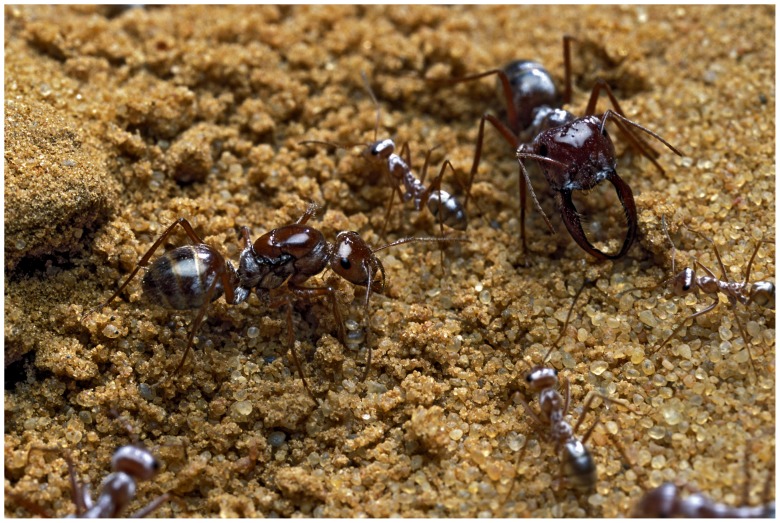
*Cataglyphis bombycina* ants are highly polyphenic. Colonies contain one dealate queen (left), numerous workers (middle and bottom), and fewer large individuals with saber-shaped mandibles ‘ISM’ (soldiers, right). Photo © P. Landmann.

In ants, phenotypic plasticity generates two morphologically distinct female castes: workers and queens. The growth rules of these adult phenotypes differ in both growth rates and critical size (when a larva reaches critical size, pupation is triggered) [1]. Queens are generally large, with a complex articulated winged thorax, and a specialized reproductive apparatus, whereas workers are smaller, with a simplified wingless thorax, and a reduced reproductive apparatus ( [2]; Fig. 1). These two castes are adapted for distinct functions, namely independent colony foundation and reproduction for queens, and foraging, brood care, nest building and nest defense for workers.

**Figure 2:**
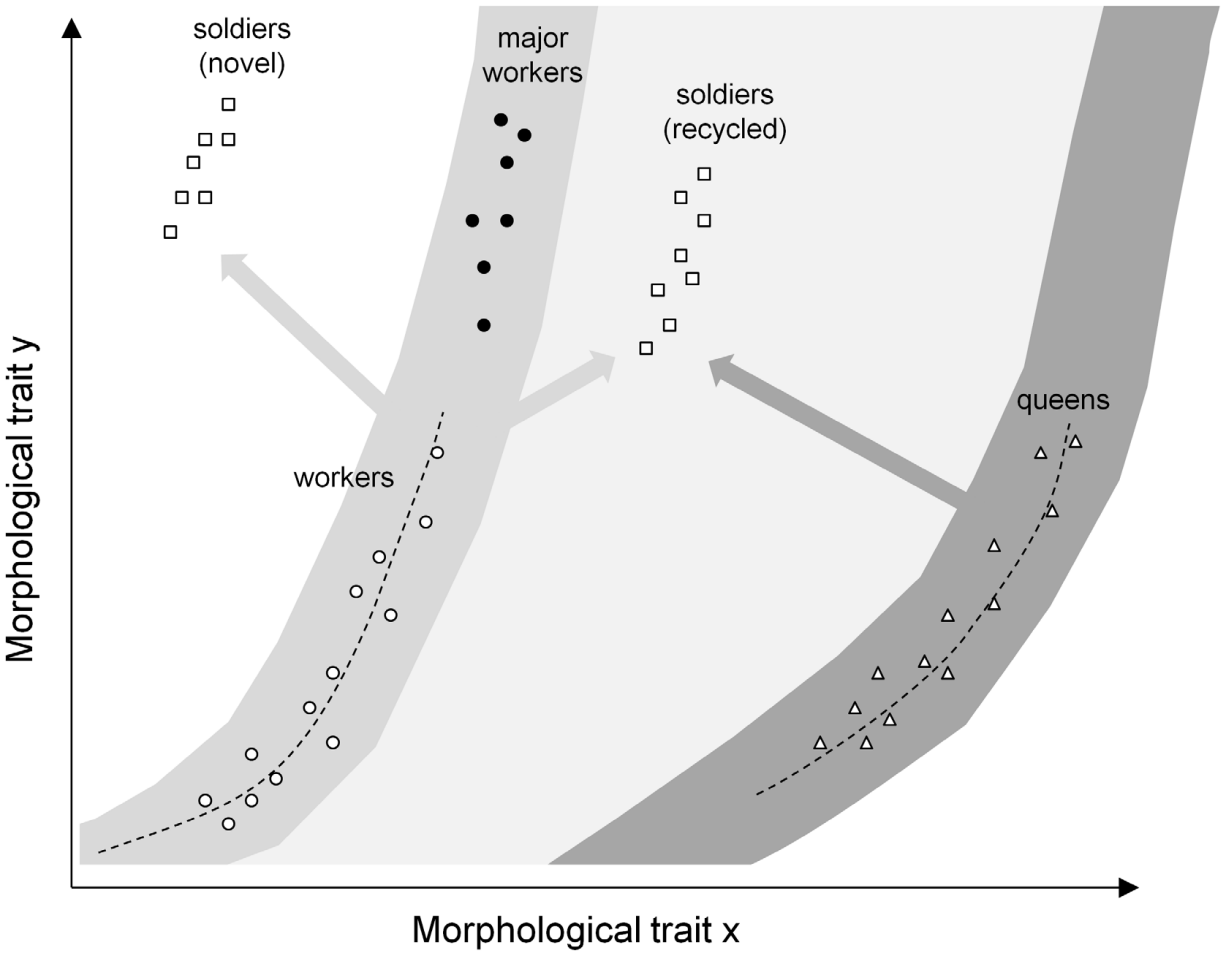
Distinct adult castes exhibit distinct growth rules. Growth rules of the worker (empty circles) and queen (empty triangles) castes for a theoretical pair of morphological traits ‘x’ and ‘y’ are illustrated by dashed lines. Increasing critical size of the worker growth rule leads to the production of adults that are larger and have a different shape due to allometry, i.e. major workers (black circles). However the range of possible phenotypes that can be produced is limited (grey area surrounding the workers’ curve). Alternatively, modifying both critical size and growth rate leads to the production of novel adult phenotypes that are outside of this range and accordingly do not belong to the worker caste, i.e. soldiers (empty squares). We suggest that this can be done either by combining parameters of the growth rules of existing worker and queen castes (‘recycled’ soldiers) or by evolving brand new growth rules (‘novel’ soldiers).

This elementary dichotomy constitutes the groundplan for all ants, but it does not reflect the diversity of female phenotypes across the 13.000 extant species. The diversification of lifestyles has selected for changes in colony life history, phenotypes of colony members and their underlying growth rules. Indeed, the degree of dimorphism between queen and worker castes has increased considerably in many taxa, and this has created an empty morphospace associated with potential new functions. For instance, size polymorphic workers can be more efficient in resource acquisition or defense, ultimately improving colony growth [3]. The production of small queens -microgynes- can be more economical for dependent colony foundation, i.e. when young queens do not fly away but disperse on foot with nestmate workers [4]. Such female phenotypes result from changes in critical size relative to the ancestral phenotypes, but not in growth rates. Indeed, major workers are distributed along the growth curve of workers, with some traits becoming enlarged relative to others due to allometry only ( [1]; Fig. 2). Similarly, microgynes in some species are ‘isometric reductions’ of macrogynes [5]. In contrast, other phenotypes result from changes in both critical size and growth rates, such as soldiers (e.g. [6]–[12]; Fig. 2) and ergatoid -permanently wingless- queens (reviewed in [13]). Their production also enhances colony success, respectively for survival/growth [7] and reproduction [14]. They can be seen as functional equivalents of major workers and microgynes respectively, although the mechanisms that produce them differ. Importantly the phenotypic outcomes of such new growth rules are potentially much more diverse than those based on similar growth rates (Fig. 2).

*Cataglyphis* ants are characteristic insects of arid regions distributed around the Mediterranean Basin and reaching into Central Asia [15]. Out of 90 species, most have continuously polymorphic workers. In contrast, *C. bombycina* (Roger) has colonies with two types of non-reproductive females: workers with variable sizes (reaction norm, i.e. continuous variation) and invariably big individuals with saber-shaped mandibles (discrete size; Fig. 1). The latter are morphologically striking and occur only in this species and *C. kurdistanicus* [16]. Their function in colonies is poorly understood. In *C. bombycina,* Délye [17] reported that they are rarely seen outside, but when the nest is disturbed they run around with open mandibles and bite.

We aimed to test whether individuals with saber-shaped mandibles (‘ISM’) are the product of allometric growth along the worker developmental curve (same growth rates but distinct critical sizes) or whether they are the outcome of completely different growth rules (new growth rates and new critical size). The first case would correspond to a major worker subcaste, whereas the second case would be a novel soldier caste. Accordingly, we compared the size, morphology and growth rules of ‘ISM’ with workers and queens. We also assessed behavior and physiology.

We discuss why the production of new phenotypes using existing growth rules is relatively risk-free in terms of viability and functionality but limits the number of possible outcomes. In contrast, the production of novel phenotypes using new growth rules can generate high diversity at the price of higher failure rate. We argue that ants can escape this cost by recombining worker and queen growth rules to produce novel phenotypes [18].

## Materials and Methods

### Colony Collection

*Cataglyphis bombycina* is a dominant species in the sand dunes of north Africa; nests are huge and deep, with multiple entrances distant by several meters. One colony (#1) was partly excavated in November 2010 by C. Peeters in Remlia, SE of Merzouga, Morocco (30.71°, −4.40°); only surface chambers (<1 m deep) could be sampled due to collapse of the rapidly drying sand. This yielded about 1000 adult ants including workers and individuals with saber-shaped mandibles, but no gynes (virgin winged queens). Four colonies (#2–5) were sampled by Serge Aron in Amerzgane (31.05°, −7.21°) [19] during nuptial flights in April 2011, and this yielded workers and gynes. No specific permissions were required to collect ants as these sand dunes are public land. Our field study did not involve endangered or protected species. Voucher specimens have been deposited in the California Academy of Sciences (see www.antweb.org for images, specimen IDs CASENT0906666, CASENT0906667, CASENT0906668).

### Data Collection

We used 48 workers (40 random, 4 of the smallest and 4 of the largest) and 40 ‘ISM’ (individuals with saber-shaped mandibles) from colony #1, and 12 random workers and 56 gynes from colonies #2–5. We measured seven body parts: head width, palp length, mandible length, area of the interior side of the mandible, tibia length, thorax volume and cross-sectional area of the first gaster segment (abdominal segment III) using ImageJ software (http://rsb.info.nih.gov/ij) following Molet et al. [20]. Since workers and gynes originated from different colonies, we first checked that the size of the various body parts did not differ between colonies. Kruskal-Wallis rank tests revealed no colony effect on either worker or queen body parts (*P*-values respectively 0.74 and 0.06 for head width, 0.19 and 0.72 for palp length, 0.60 and 0.06 for mandible length, 0.53 and 0.06 for area of the interior side of the mandible, 0.50 and 0.88 for tibia length, 0.51 and 0.06 for thorax volume and 0.21 and 0.69 for cross-sectional area of the first gaster segment. We also took scanning electron microscope photographs of all female types.

### Statistical Analyses

In order to contrast growth rules among female types, we compared the sizes of body parts and the growth rates between body parts. Sizes were compared using Kruskal-Wallis rank tests followed by pairwise comparisons using Wilcoxon rank tests with Bonferroni correction. Growth rates, also known as allometry coefficients, were computed as follows. First we homogenized our measures of area and volume to a single linear dimension (equivalent to a length) using square and cube root transformations respectively. Second, we log-transformed the homogenized length, area and volume data. Third, we performed a correlation analysis using pairs of transformed variables, and if it was significant we computed a regression line between Y and X variables, the slope of which is the allometry coefficient (growth rate). This coefficient describes how much body part Y grows when body part X grows. When the allometry coefficient equals one, body parts grow at the same rate so body shape does not change with size (isometry). When it differs from one, body parts grow at a different rate so body shape changes with size (allometry). For instance, a coefficient of three means that Y growths at a cubic rate relative to X, i.e. Y = X^3^. Accordingly, when X gets bigger, Y gets much bigger and body shape changes dramatically. We tested whether the allometry coefficient differed from one (allometry) or not (isometry). Finally, we determined whether queens, workers and ‘ISM’ exhibit different growth rates by comparing their allometry coefficients for all pairs of body parts. We also compared elevations, which reflect critical size and are related to the intercepts of the regression lines. Allometry coefficients and elevations were computed and compared using (S)MATR 1.0 [21] (http://www.bio.mq.edu.au/ecology/SMATR/). All other statistical analyses were performed with R 2.13 (available at http://cran.r-project.org/).

Among workers, size distribution was continuous, so we treated them as a single group. Among queens however, size distribution of most body parts was bimodal (Fig. S1). Two queen castes with distinct sizes and growth rules may thus exist, so pulling them together may result in incorrect estimates and erroneous comparisons with ‘ISM’. Accordingly, before performing any comparisons among female types, we split queens in two groups: macrogynes and microgynes. ‘ISM’ were thus compared to workers, macrogynes and microgynes, to test two hypotheses: either they belong to the worker caste, or they are a third caste.

## Results

### A Distinct Size from Other Castes

**Figure 3:**
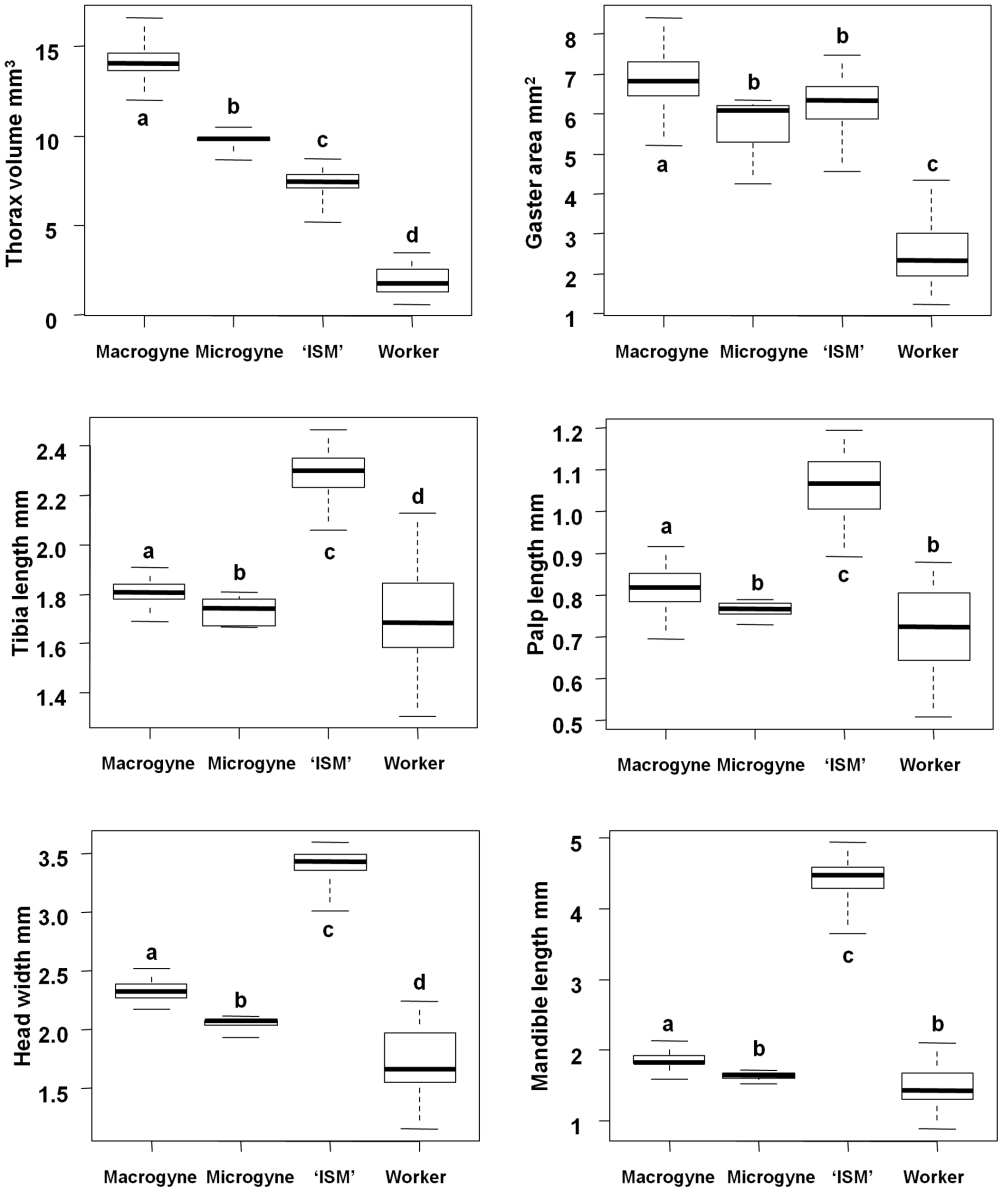
Size of body parts differs between female types in most cases. (52 workers, 47 macrogynes, 9 microgynes and 40 individuals with saber-shaped mandibles ‘ISM’). All Kruskal-Wallis rank tests are significant (*P*<0.001), and 38 out of 42 pairwise comparisons using Wilcoxon rank tests with Bonferroni-corrected *P*-values are significant (different letters indicate significant differences).

Size polymorphism among workers is considerable in *C. bombycina*: the biggest worker's head was 194% wider than the smallest worker's. We compared the size of body parts between female types, and Kruskal-Wallis tests revealed significant differences in size between female types for all body parts (P<0.001). ‘ISM’ had larger tibia, palp, head and mandible than the three other female types (Fig. 3). Their thorax volume and gaster area were intermediate between queens and workers (gaster area did not differ significantly with microgynes). Mandibles exhibited the most striking size differences among female types: saber-shaped mandibles were 3.2 times longer than workers’ mandibles and 2.4 times longer than macrogynes’.

### A Worker-like Thorax but Novel Mandibles

**Figure 4:**
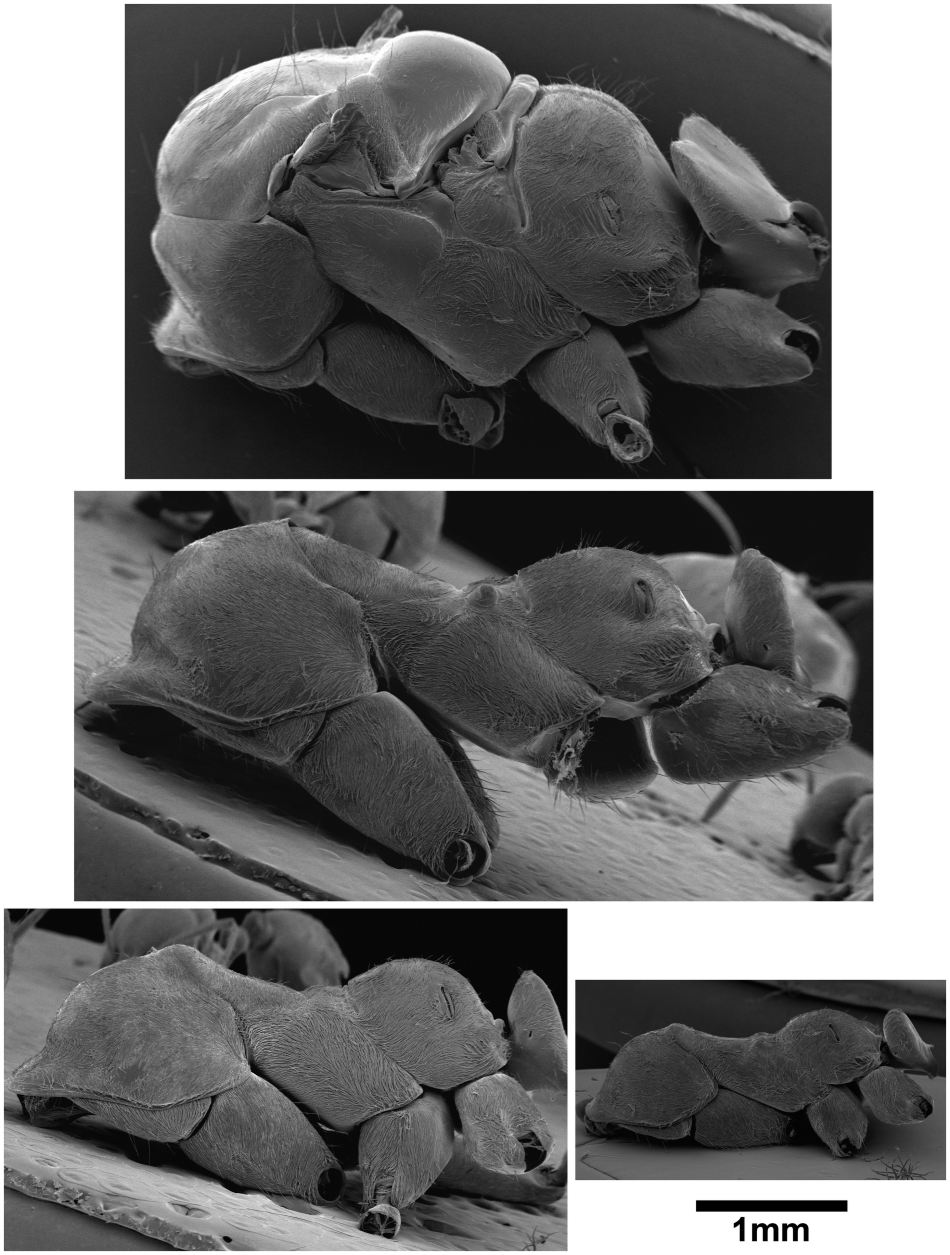
Scanning electron micrographs of the thorax of macrogyne, individual with saber-shaped mandibles ‘ISM’, large worker and small worker. (Resp. top, middle, bottom left and bottom right). The thorax of ‘ISM’ is simplified as in workers: sclerites are fused and mesonotum is reduced.

**Figure 5:**
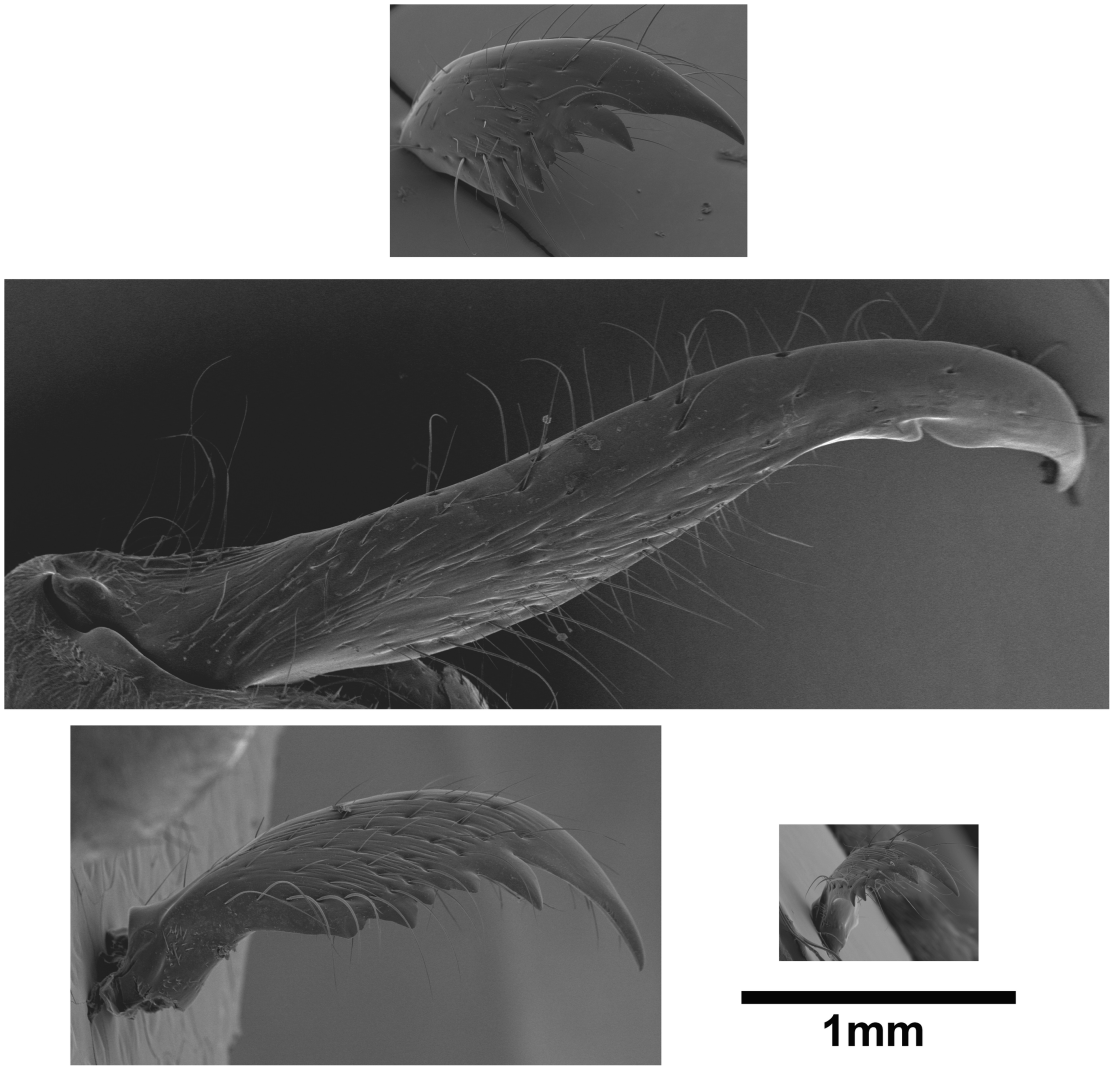
Scanning electron micrographs of the mandibles of macrogyne, individual with saber-shaped mandibles ‘ISM’, large worker and small worker. (Resp. top, middle, bottom left and bottom right). Macrogynes and workers have similar mandibles, but those of ‘ISM’ have a completely different shape and size.

Scanning electron microscopy revealed no noticeable difference in thorax structure between ‘ISM’ and workers (Fig. 4). The pronotum was prominent, while mesonotum and metanotum were fused. No articulated sclerites or marked grooves were visible. Macrogynes and microgynes differed strongly due to their flight thorax: pronotum was smaller, mesonotum was very developed (it functions to attach wing muscles) and distinct from the metanotum. Sclerites were articulated and separated by grooves. ‘ISM’ had elongated mandibles with few teeth that strongly differ in shape from the short mandibles with six teeth found in macrogynes and workers (Fig. 5).

### A Mix of Queen and Worker Growth Rates

**Figure 6:**
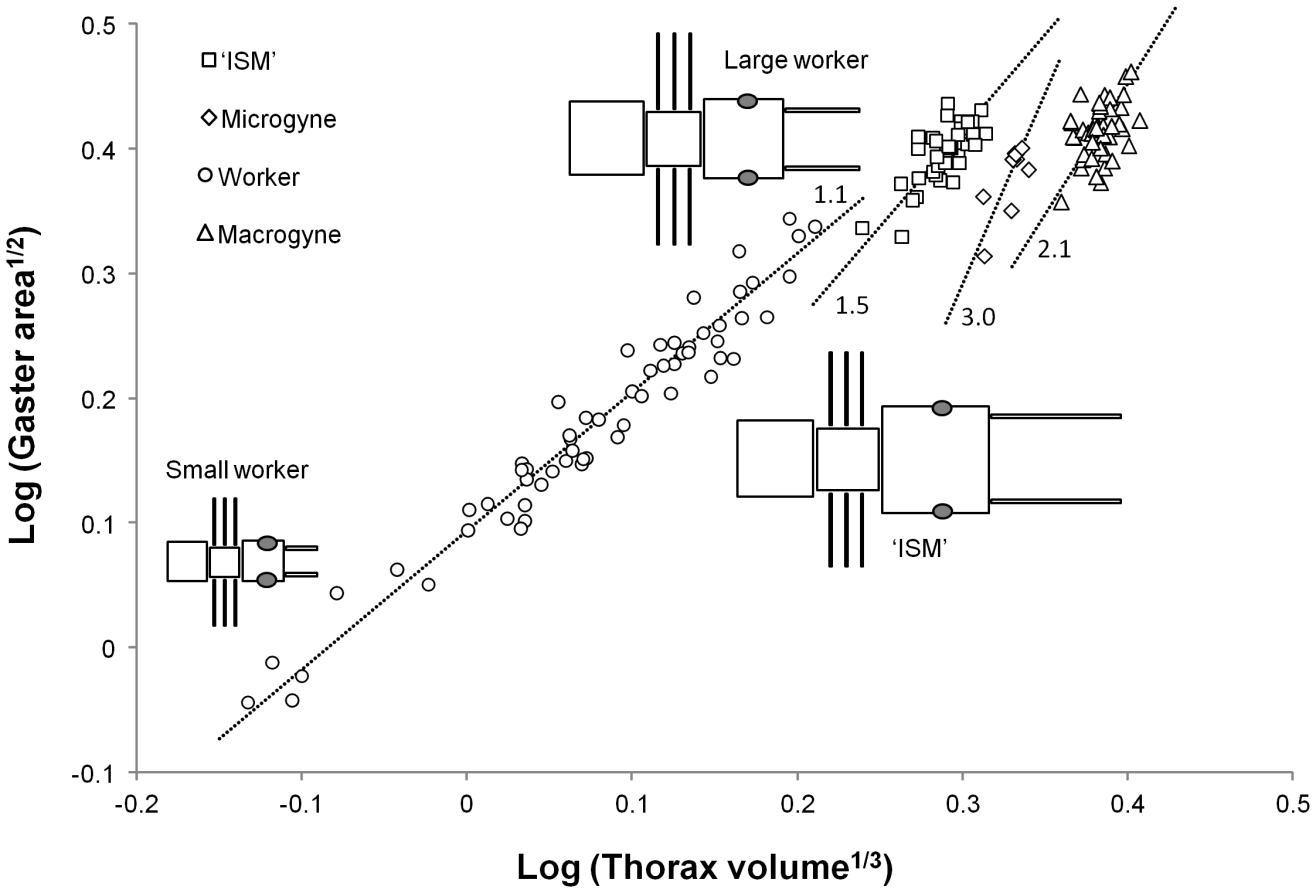
Growth rules of the four female types for one pair of morphological traits. (See Table 1 for some of the other pairs). Circles, squares, diamonds and triangles represent workers, individuals with saber-shaped mandibles ‘ISM’, microgynes and macrogynes respectively. Dotted lines are major axis regression lines, with their slopes (allometry coefficients) indicated as numbers. These four growth rules differ significantly. However, the allometry coefficients (growth rates) of ‘ISM’ and microgynes are not significantly different, suggesting that ‘ISM’ recycle a queen-like developmental program for this pair of traits. Diagrams represent a small worker, a theoretical large worker of tibia length t produced by the allometric growth rules of the worker caste, and an ‘ISM’ of the same tibia length t produced by the new growth rules. The large worker and the ‘ISM’ clearly differ. This illustrates that worker growth rules cannot produce a major worker phenotype that would look like an ‘ISM’ (Fig. 2).

The sizes of several pairs of body parts in some female types were not significantly correlated. Accordingly, out of the 21 growth rules available, we used the eight growth rules that were significant for at least three female types, in order to compare them fully (Table 1, Fig. 6). The allometry coefficient of ‘ISM’ was not different from that of workers but different from that of macrogynes and microgynes in one rule out of eight. In contrast, it was not different from that of macrogynes but different from that of workers and microgynes in six rules out of eight. Accordingly, ‘ISM’ were more similar to macrogynes than to workers or microgynes regarding allometry coefficients (growth rates). However, in all cases where allometry coefficients did not differ between female types, elevation of the regression lines differed, indicating a distinct critical size in ‘ISM’.

**Table 1:** Growth rules of the four female types differ, revealing that they are distinct morphological castes.

	Correlation		Allometry coef	Test against isometry			Elevation
	*R^2^*	*P*	and test	*F*	*P*	Conclusion	test
Workers (*N* = 60)							
Thorax volume vs. Head width	0.98	<0.001	0.85 a	70.2	<0.001	Allometry	a
Thorax volume vs. Mandible length	0.93	<0.001	0.75 a	69.4	<0.001	Allometry	–
Thorax volume vs. Mandible area	0.96	<0.001	0.80 a	78.1	<0.001	Allometry	a
Gaster area vs. Thorax volume	0.95	<0.001	1.11 a	13.2	0.001	Allometry	–
Gaster area vs. Head width	0.95	<0.001	0.94 a	3.5	0.066	Isometry	–
Mandible length vs. Head width	0.94	<0.001	1.14 a	16.5	<0.001	Allometry	–
Mandible length vs. Mandible area	0.98	<0.001	1.06 a	11.4	0.001	Allometry	a
Mandible area vs. Head width	0.97	<0.001	1.07 a	8.5	0.005	Allometry	a
Macrogynes (*N* = 47)							
Thorax volume vs. Head width	0.39	<0.001	0.68 a	11.9	0.001	Allometry	b
Thorax volume vs. Mandible length	0.12	0.016	0.42 b	50.7	<0.001	Allometry	a
Thorax volume vs. Mandible area	0.32	<0.001	0.65 a	13.2	0.001	Allometry	b
Gaster area vs. Thorax volume	0.18	0.003	2.09 bc	35.4	<0.001	Allometry	a
Gaster area vs. Head width	0.15	0.007	1.41 b	6.6	0.013	Allometry	b
Mandible length vs. Head width	0.31	<0.001	1.63 b	16.7	<0.001	Allometry	b
Mandible length vs. Mandible area	0.50	<0.001	1.56 b	18.9	<0.001	Allometry	–
Mandible area vs. Head width	0.33	<0.001	1.04 ab	0.1	0.73	Isometry	b
Microgynes (*N* = 9)							
Thorax volume vs. Head width	0.66	0.008	0.80 a	1.0	0.35	Isometry	b
Thorax volume vs. Mandible length	0.11	0.39	–	–	–	–	–
Thorax volume vs. Mandible area	0.07	0.51	–	–	–	–	–
Gaster area vs. Thorax volume	0.57	0.019	3.03 b	29.8	0.001	Allometry	b
Gaster area vs. Head width	0.26	0.16	–	–	–	–	–
Mandible length vs. Head width	0.19	0.24	–	–	–	–	–
Mandible length vs. Mandible area	0.01	0.79	–	–	–	–	–
Mandible area vs. Head width	0.01	0.76	–	–	–	–	–
Individuals with Saber-shaped Mandibles ‘ISM’ (*N* = 40)							
Thorax volume vs. Head width	0.58	<0.001	0.87 a	1.8	0.19	Isometry	c
Thorax volume vs. Mandible length	0.14	0.017	0.52 b	21.5	<0.001	Allometry	b
Thorax volume vs. Mandible area	0.34	<0.001	0.61 a	15.1	<0.001	Allometry	c
Gaster area vs. Thorax volume	0.55	<0.001	1.54 c	16.7	<0.001	Allometry	c
Gaster area vs. Head width	0.39	<0.001	1.34 b	5.4	0.026	Allometry	c
Mandible length vs. Head width	0.37	0.023	1.67 b	17.2	<0.001	Allometry	c
Mandible length vs. Mandible area	0.59	<0.001	1.17 a	2.3	0.14	Isometry	b
Mandible area vs. Head width	0.65	<0.001	1.43 b	14.0	0.001	Allometry	c

Column #1: pair of morphological traits for which relative growth rule was assessed (Y vs. X). #2: Pearson correlation test. #3: slope of the regression line (allometry coefficient) and comparison test between female types. #4: comparison of the slope with 1 (isometry). #5: when slopes do not differ between female types, comparison of elevations between female types. Pairs of morphological traits that were not significantly correlated at the same time in three female types were excluded. Thus only eight growth rules are shown out of 21 potential growth rules. Correlations are rarely significant in microgynes due to small sample size.

## Discussion

### A Soldier Caste, not Major Workers

Our morphometric data show that the large non-reproductive individuals in *C. bombycina* do not follow the growth rules of workers. Their shape cannot be produced by a simple change in critical size along the worker growth curve (Fig. 6). Thus they are a soldier caste according to our definition (Introduction and [18]), and not major workers (Fig. 2). They do not follow the growth rules of queens either. *C. bombycina* is an ideal model to study the evolution of morphological diversity among sterile individuals. Indeed, this species has a highly polymorphic worker caste and a monomorphic soldier caste. This contrasts with what is known in other species. For instance, *Camponotus festinatus* has a polymorphic worker caste and a polymorphic soldier caste [1], whereas *Pheidole* species and *Atta texana* have a monomorphic worker caste (‘minors’) and one or two distinct soldier castes [22], [23]. Phenotypic diversity within a colony can thus be achieved using multiple developmental options, i.e. by producing novel soldier castes and/or by generating various degrees of intra-caste variability. Our description of unambiguous morphological differences between individuals with saber-shaped mandibles and large/major workers supports the restrictive definition of soldiers advocated by Molet et al. [18], i.e. soldiers are a third caste, neither workers nor queens.

### A Mix of Worker and Queen Developmental Programs

New phenotypes require changes in growth rules, and these are caused by changes in gene expression during development. This requires either new mutations or environmental release of cryptic genetic variation [24]. In monomorphic organisms, these new growth rules may lead to the production of lethal or useless phenotypes, and selection over many generations may be required to reach a well-adapted phenotype. In contrast, polyphenic organisms already have distinct sets of growth rules corresponding to each type of phenotype. These growth rules have been selected for and they produce viable phenotypes. Instead of evolving new growth rules, polyphenic organisms may re-utilize existing growth rules and recombine them to produce new phenotypes [18]. In ants, soldiers and ergatoid (permanently wingless) queens may be the product of such a mechanism.

In *C. bombycina*, we found that the allometry coefficients (growth rates) of soldiers were never different from either worker or queen castes. Instead, they were sometimes similar to workers, and sometimes similar to queens. Thus, soldiers do not exhibit new growth rates, but they recombine growth rates from both worker and queen castes. Hence their production relies on recycling (Fig. 2), and we argue that the ancestral queen-worker polyphenism contributed to the evolution of a novel female phenotype in this species.

In contrast, the elevations of regression lines of soldiers always differed from both worker and queen castes so growth rules were not fully conserved: growth rates were, but critical sizes were not. Moreover, it is not clear how changes in gene expression relative to workers and queens can lead to the modified mandibles of soldiers. 2D morphometrics would be required to assess deformations in mandible shape and discuss the developmental link between these mandible types. Future studies in evo-devo should investigate how much recycling is involved in the production and evolution of novel traits in ants, and whether this recycling could explain the numerous independent evolutions of novel castes across the ant phylogeny.

### A Specialization for Defence and Food Storage

Soldiers have saber-shaped mandibles (together with a broad head with powerful muscles) that are similar to those of soldiers in army ants (e.g. *Eciton*; [25]); these are thought to be adapted for defense against vertebrate predators, not arthropods. Accordingly, *C. bombycina* soldiers may be specialized for defense against reptiles. *C. bombycina* is the dominant ant species in the harsh sand dune habitats of North Africa. Their colonies are exceptionally populous for this genus, and the large quantities of brood developing in the deep underground chambers are likely to represent a valuable resource for reptiles. Lizards in the genus *Acanthodactylus* are known to be the main predators of *C. bombycina* [26], and it is possible that they dig ant nests to find brood. This hypothesis is corroborated by the finding of numerous soldiers in deep brood chambers (S. Aron pers. com.). In this species, soldiers are only useful inside the nest because there is no compact resource to defend outside: food items are scattered insect corpses collected up to 100 m away from the nest. Colonies of other *Cataglyphis* species are much smaller and lack a soldier caste [15], [27]. Cagniant [27] suggested that soldiers are produced only in older (i.e. bigger) colonies of *C. bombycina.* Unlike Délye [17], we found that soldiers have longer palps than the larger workers, and this could allow them to carry bigger sand pellets [28].

The abdomen of some soldiers and major workers kept in the laboratory with *ad libitum* food was conspicuously distended. Dissections revealed large accumulations of fat bodies. Workers functioning as repletes have been described in other *Cataglyphis* species [27]. *C. bombycina* soldiers have an abdomen with a cross-sectional area that is 2.7 times larger than workers', thus allowing for much more storage in a soldier than in a worker. Accordingly, we suggest that soldiers are also specialized to function as repletes; a similar function (production of trophic eggs) was shown for the soldier caste in *Crematogaster (Orthocrema)* [29]. Many ant species store excess food that is later shared among nestmates [30]. Storage is usually carried out by workers, but some specialized castes have evolved in some species. The latter do not follow the growth rules of workers, and they have queen-like ovaries, hence they fall into our definition of ‘soldiers’. *C. bombycina* feeds on dead insects which are an unpredictable resource, so food storage is likely to be adaptive for colonies. It is not clear whether soldiers initially evolved for defense or food storage, however this dual function balances their production cost. Future studies need to determine whether young soldiers function as repletes in deep chambers and older soldiers move to surface chambers to be available for nest defense.

### A Second Queen Caste?

Although not the primary focus of our study, we found that *C. bombycina* has dimorphic queens: macrogynes and microgynes. Microgynes were too few to assess their growth rules and compare them to macrogynes, workers and soldiers. In contrast with soldiers, microgynes in *C. bombycina* may not result from a mosaic development, and an increased sample size is required to test whether or not microgynes are mosaics of macrogynes and workers. Having microgynes in addition to macrogynes can be advantageous for colonies. Macrogynes typically perform independent colony foundation, i.e. they fly away from their natal nest, mate, and start a new colony alone. In contrast, microgynes can stay in their natal nest where they reproduce (polygyny), and/or be involved in dependent colony foundation (fission). In some species, microgynes enter nearby colonies and become social parasites. Accordingly, microgynes are thought to be an adaptation to saturated habitats with high competition [4]. Since they are less costly to produce relative to macrogynes, more of them can be produced with the same amount of resources. The function of microgynes of *C. bombycina* is unknown.

### Colonial Life Facilitates the Evolution of New Phenotypes

Our morphometric analysis showed that polyphenic taxa can produce novel phenotypes by recombining growth rules from existing phenotypes. Such mosaics are probably more frequent and viable than new mutants. This original developmental mechanism could enhance the evolvability of polyphenic species, and it may have contributed to the tremendous diversification of ants. In addition, phenotypes that would be suboptimal or lethal in a solitary context can survive in ants because colonies buffer the outside environment. Accordingly, selection for new mosaic phenotypes can be facilitated provided they bring colony-level benefits for defense, food storage or reproduction [18]. The properties of social life as an incubator for evolutionary novelties should be considered in future research.

## Supporting Information

Figure S1 **Distribution of thorax volume across female types reveals queen bimodality with microgynes and macrogynes.**
(TIF)
